# Synthesis and targeting of gold-coated ^177^Lu-containing lanthanide phosphate nanoparticles—A potential theranostic agent for pulmonary metastatic disease

**DOI:** 10.1063/1.5018165

**Published:** 2017-12-27

**Authors:** Nicholas Sobol, Logan Sutherlin, Edyta Cedrowska, Joshua Schorp, Cristina Rodríguez-Rodríguez, Vesna Sossi, Jimmy Lattimer, Douglas C Miller, Paul Pevsner, J. David Robertson

**Affiliations:** 1 Department of Chemistry, University of Missouri, Columbia, Missouri 65211, USA; 2 University of Missouri Research Reactor (MURR), Columbia, Missouri 65211, USA; 3 Institute of Nuclear Chemistry and Technology, Dorodna 16, 03-195 Warsaw, Poland; 4 Department of Physics and Astronomy, University of British Columbia, Vancouver, British Columbia V6T 1Z1, Canada; 5 Faculty of Pharmaceutical Science, University of British Columbia, Vancouver, British Columbia V6T 1Z3, Canada; 6 Centre for Comparative Medicine, University of British Columbia, Vancouver, British Columbia V6T 1Z3, Canada; 7 Department of Veterinary Medicine and Surgery, University of Missouri, Columbia, Missouri 65211, USA; 8 Department of Pathology and Anatomical Sciences, University of Missouri School of Medicine, Columbia, Missouri 65212, USA; 9 Nano Imrad Technology, Inc., Irving, Texas 75039, USA

## Abstract

Targeted radiotherapies maximize cytotoxicity to cancer cells. In this work, we describe the synthesis, characterization, and biodistribution of antibody conjugated gold-coated lanthanide phosphate nanoparticles containing ^177^Lu. [^177^Lu]Lu_0.5_Gd_0.5_(PO_4_)@Au@PEG_800_@Ab nanoparticles combine the radiation resistance of crystalline lanthanide phosphate for stability, the magnetic properties of gadolinium for facile separations, and a gold coating that can be readily functionalized for the attachment of targeting moieties. In contrast to current targeted radiotherapeutic pharmaceuticals, the nanoparticle-antibody conjugate can target and deliver multiple beta radiations to a single biologically relevant receptor. Up to 95% of the injected dose was delivered to the lungs using the monoclonal antibody mAb-201b to target the nanoparticles to thrombomodulin receptors. The 208 keV gamma ray from ^177^Lu decay (11%) can be used for SPECT imaging of the radiotherapeutic agent, while the moderate energy beta emitted in the decay can be highly effective in treating metastatic disease.

## INTRODUCTION

Nanoparticle systems are being developed extensively around the world for applications in cancer imaging and therapeutics. Their ability to contain radioactive atoms and specifically target cancer cells with the use of peptides or antibodies makes them appealing candidates as targeted radiotherapy agents.

The use of targeted radiotherapy is becoming more widespread. Examples include drugs such as Xofigo^®^ (RaCl_2_) for the treatment of bone metastases in patients with castration-resistant prostate cancer and Zevalin^®^ (Yttrium-90 ibritumomab tiuxetan) for the treatment of non-Hodgkin's lymphoma. In the case of Xofigo, targeting is accomplished because radium behaves as a calcium mimic, whereas in the case of Zevalin, targeting is achieved through the use of an antibody. A promising targeted radiotherapeutic in development is Lutathera^®^, a ^177^Lu radiolabeled somatostatin analog that targets somatostatin receptors, which are over-expressed in several types of cancer. As of August 2016, Lutathera has received a priority review from the U.S. Food and Drug Administration (FDA) after the results of a phase III clinical trial showed a 79% reduction in disease progression or death in the treatment of gastroenteropancreatic neuroendocrine tumors as compared to Octreotide acetate (Sandostatin^®^ LAR Depot).^1–3^ In each of these targeted radiotherapy approaches, the targeting agent delivers, at most, one radioactive atom per targeted receptor site.

Nanoparticle-based drug delivery has the potential to provide several key advantages over traditional targeted radiotherapy treatments. Using a nanoconjugate, which consists of a nanoparticle, a linking agent, and an antibody or a peptide, it is possible to combine many useful properties including stabilizing the radiometal in the nanoparticle, tuning of the biodistribution through modification of the surface of the nanoparticle, and, perhaps most importantly, the ability to deliver more than one copy of a radiotherapeutic to a single receptor site. Delivering multiple radioactive atoms per receptor will increase the cytotoxicity of nanoparticle based agents as compared to currently approved targeted radiotherapies. Modular surface modification allows for the tailored biodistribution of the nanoparticle system to maximize accumulation at the tumor site. Arvizo *et al.* have demonstrated the effect of changing the surface charge of gold-coated nanoparticles by using positively charged, negatively charged, neutral, and zwitterionic head groups.^4^ The neutral and zwitterionic particles exhibit higher peak plasma concentrations, lower plasma clearance, longer blood circulation time, and higher tumor uptake than their charged counterparts. Moreover, the authors have demonstrated successful receptor-mediated targeting of lanthanide phosphate nanoparticles loaded with the *in vivo* alpha generator ^225^Ac.^5,6^ Using the monoclonal antibody mAb-201b, which targets thrombomodulin receptors in the lungs, we demonstrated antibody-mediated uptake of 30% of an intravenously injected dose (ID) in the lungs 1-h post-injection.^7^ With the use of clodronate liposomes, which deplete the number of macrophages available to clear particulates such as nanoconjugates from the bloodstream, the amount of injected dose in the lungs rose to 47%. The same study also demonstrated a significant reduction in EMT-6 tumor colonies in the lungs in mice following a single IV injection of the lung-targeted ^225^Ac nanoparticle conjugates.

In this work, we describe the synthesis, characterization, and biodistribution of antibody conjugated gold-coated lanthanide phosphate nanoparticles containing ^177^Lu. The nanoparticles are modular and contain several different and useful moieties. They combine the radiation resistance of crystalline lanthanide phosphate for stability, the magnetic properties of gadolinium for facile separations, and a gold coating that can provide the ability to functionalize the surface with well-established gold-surface chemistry and the ability to incorporate multiple atoms of the beta-emitting isotope ^177^Lu. In contrast to current work in which ^177^Lu is attached to the surface of gold^8,9^ or polymeric nanoparticles,^10^ the radioactive isotope in the lanthanide phosphate nanoparticles is bound within the crystalline core, enhancing the *in vivo* stability of the construct.

While a plethora of potentially useful nanoparticle delivery systems have been developed in labs across the world, specifically targeting them to receptors *in vivo* has proven to be more challenging. In a recent review covering the last 10 years of nanoparticle delivery to solid tumors, it was found that only 0.7% (median) of an administered nanoparticle dose will reach its target. The nanoconjugates described here demonstrate the ability to specifically target up to 95% injected dose (ID) to their target antigens, which is a dramatically higher delivery efficiency than the recently reported median.^11^

The objective of the work reported herein is to expand upon previous work with ^225^Ac nanoconjugates by using ^177^Lu to target metastatic cancer in the lungs. Pulmonary metastatic disease is the most common form of secondary lung tumors, which are identified in 30%–55% of all cancer patients. Lungs are the principle site of metastasis in breast, colon, and pancreatic cancers, representing a common site of treatment failure. The presence of multifocal pulmonary metastatic disease is not amenable to surgical resection, and the disease burden often limits the effectiveness of chemotherapy. By using nanoparticles that contain alpha and beta emitters, we will be able to tailor the treatment with short-range (∼0.05 mm) high linear energy-transfer radiation (alpha) and longer range (∼3 mm) lower linear-energy transfer radiation (beta) to improve our ability to deliver localized therapy to metastatic cancer.

## RESULTS AND DISCUSSION

### Nanoparticle characterization

**FIG. 1.**
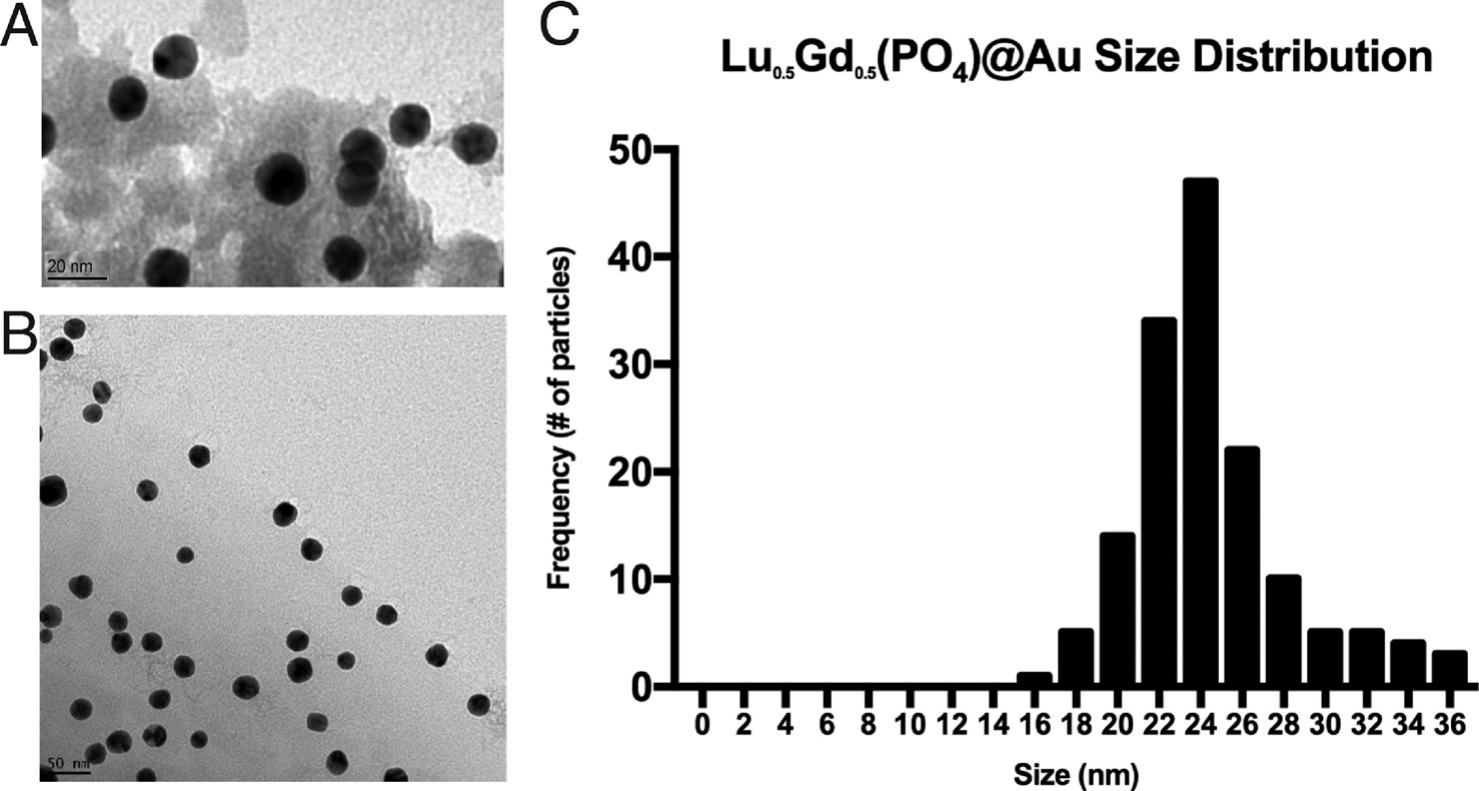
(a) TEM image of core Lu_0.5_Gd_0.5_(PO_4_) nanoparticles. (b) TEM image of Lu_0.5_Gd_0.5_(PO_4_)@Au gold-coated nanoparticles. (c) Lu_0.5_Gd_0.5_(PO_4_)@Au gold-coated nanoparticle size distribution.

Nanoparticle cores [Lu_0.5_Gd_0.5_(PO_4_)] analyzed using transmission electron microscopy (TEM) had an average diameter of 16 ± 2.1 nm [Fig. 1(a)]. Neutron activation analysis results showed the composition of Gd and Lu in the core to be 47.5 ± 1.1 mol. % lutetium and 52.5 ± 1.1 mol. % gadolinium. Other core compositions were synthesized and analyzed by neutron activation analysis (NAA), and their compositions are given in Table I.

**TABLE I.** Metal composition (mol. %) of nanoparticles.

Nanoparticle	Lu	Gd	Au
Lu_0.5_Gd_0.5_(PO_4_) (n = 3)	47.5 ± 1.0	52.5 ± 1.1	N/A
Lu_0.5_Gd_0.5_(PO_4_)@Au (n = 3)	43.2 ± 1.7	56.7 ± 1.7	0.08 ± 0.002
Lu_0.25_Gd_0.75_(PO_4_)@Au	20.3	79.6	0.07
Lu_0.1_Gd_0.9_(PO_4_)@Au	8.5	91.4	0.07
Lu_0.75_Gd_0.25_(PO_4_)@Au	71.5	28.4	0.05
Lu_0.9_Gd_0.1_(PO_4_)@Au	88.4	11.4	0.17

Gold-coated nanoparticles [Lu_0.5_Gd_0.5_(PO_4_)@Au)] analyzed using TEM had an average diameter of 23.5 ± 3.8 nm [Figs. 1(b) and 1(c)] and exhibited a plasmon resonance band at 529 nm in the ultraviolet-visible (UV-vis) spectrum. UV-vis spectroscopy was also used to monitor the addition of the polyethylene glycol (PEG) linker to the surface of the nanoparticles. Changes in the refractive index surrounding the nanoparticles caused a shift in the plasmon resonance band. This shift confirmed the binding of PEG linkers or antibodies to the surface of the nanoconjugates.^12^ The gold-coated nanoconjugate plasmon band blue shifted from 529 nm to 525 nm with PEG addition. Adding the antibody shifted the plasmon band from 525 nm to 536 nm.

### Radiochemical yield and stability

The average amount of ^177^Lu incorporated into the core nanoparticles during multiple syntheses (n = 10) was 95 ± 2.8%. The ability of the core [^177^Lu]Lu_0.5_Gd_0.5_(PO_4_) (non-gold-coated) particles to retain ^177^Lu was determined by challenging the cores with 18 MΩ water. Over a period of two weeks, the cores lost 30% of the ^177^Lu activity. Because of the very low K_sp_ values for lanthanide phosphate crystals (10^−26^–10^−27^), we hypothesized that this decrease in ^177^Lu retention was not a result of leeching from the nanoparticle core but was release of surface bound ^177^Lu cations. To test this hypothesis, an exchange experiment where an excess amount of cold LuCl_3_ salt was added to the solution during the synthesis of the core nanoparticles was performed. The exchange lutetium was allowed to mix vigorously with the core nanoparticles for 24 h. The nanoparticles were centrifuged, and the supernatant was decanted. In this experiment, the cores lost 21% of their activity in 24 h, which corresponded to the loss seen over a week when the particles were not challenged with the exchange material (supplementary material, Fig. 1). While not conclusive, this result supports the hypothesis that loss of ^177^Lu from the core is due to surface activity and not nanoparticle dissolution.

The overall radiochemical yield of ^177^Lu incorporated in the [^177^Lu]Lu_0.5_Gd_0.5_(PO_4_)@Au nanoparticles was 66.5 ± 4.7% (n = 10). This value was determined by measuring the activity of the magnetically separated nanoparticles following gold-coating. Over a time period of 17 days in 18 MΩ water, the gold-coated nanoparticles retained more than 95% of the ^177^Lu activity and in phosphate-buffered saline (PBS), they retained more than 98% of ^177^Lu. In fetal bovine serum (FBS) at 37 °C, the gold-coated nanoparticles retained more than 95% of the ^177^Lu activity (supplementary material, Fig. 2).

### *In vivo* biodistribution of PEG coated nanoparticles

The effect of the PEG linker length on the biodistribution of the radiolabeled Lu_0.5_Gd_0.5_(PO_4_)@Au@PEG nanoparticles was investigated by changing the length of the PEG linker from 800 to 5000 Da. Three differently sized PEG linkers [molecular weight (MW) = 800, 3500, and 5000 Da] were attached to the nanoparticle surface, and the resulting nanoconjugates were dispersed in 18 MΩ water and allowed to sit. The nanoparticles conjugated to the 800 Da PEG settled out of solution after only 1 h, while those conjugated to the 3400 and 5000 Da PEG settled in about 24 to 48 h. These observations qualitatively support the known phenomena, whereby increasing the PEG linker length imparts greater stability to nanoparticles in solution by preventing aggregation. In each case, the nanoparticles could be readily re-dispersed in solution with simple vortex mixing. The hydrodynamic diameters of all three conjugates determined by dynamic light scattering (DLS) are given in Table II.

**TABLE II.** Hydrodynamic diameter of PEG coated nanoparticles.

Nanoparticle	Hydrodynamic diameter (nm)
Lu_0.5_Gd_0.5_(PO_4_)@Au@PEG_800_	34.4
Lu_0.5_Gd_0.5_(PO_4_)@Au@PEG_3400_	40.5
Lu_0.5_Gd_0.5_(PO_4_)@Au@PEG_5000_	52.2
Lu_0.5_Gd_0.5_(PO_4_)@Au@PEG_800_@Ab	65.5

**FIG. 2.**
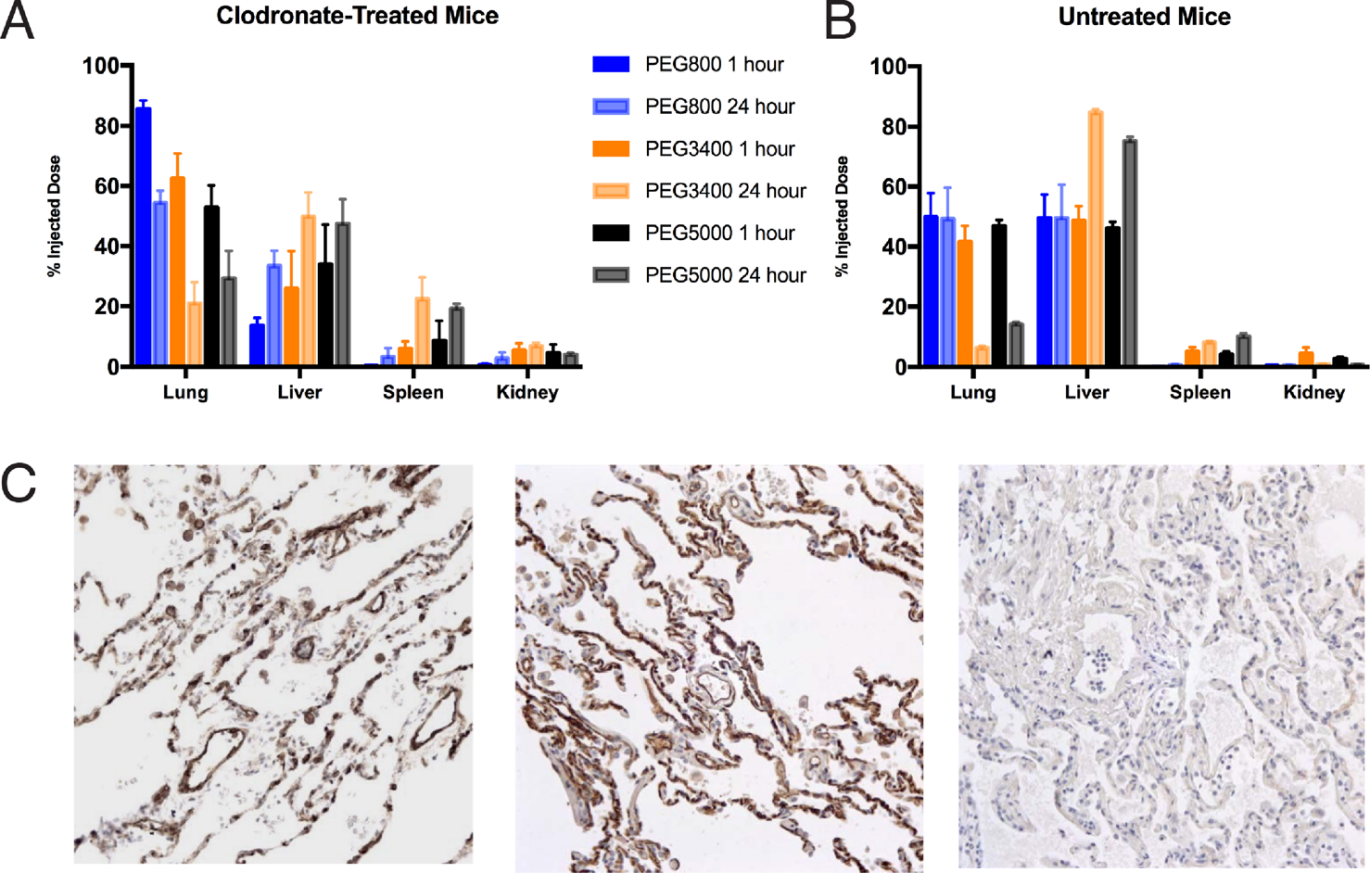
(a) Biodistribution of [^177^Lu]Lu_0.5_Gd_0.5_(PO_4_)@Au@PEG_x_ (x = 800, 3400, or 5000) in mice treated with clodronate liposomes. (b) Biodistribution of [^177^Lu]Lu_0.5_Gd_0.5_(PO_4_)@Au@PEG_x_ in untreated mice. (c) Immunohistochemistry results for Lu_0.5_Gd_0.5_(PO_4_)@Au@PEG_800_@Ab (Ab= anti-thrombomodulin) at 200× magnification. (left) An immunostain using the anti-thrombomodulin antibody as the primary antibody labels blood vessels in pulmonary alveolar septae (brown reaction product). (center) Anti-thrombomodulin-conjugated nanoparticles used instead of the primary antibody demonstrate identical immunoreactivity. (right) A negative control (no primary antibody) shows no staining in lung tissue.

The [^177^Lu]Lu_0.5_Gd_0.5_(PO_4_)@Au@PEG_800_ nanoconjugate with the shortest PEG linker (800 Da) preferentially accumulated in the lungs with 85% ID found in that organ 1-h post-injection in clodronate-treated mice [Fig. 2(a)]. The majority of remaining activity was found in the liver at 1 h. Over the course of 24 h, the [^177^Lu]Lu_0.5_Gd_0.5_(PO_4_)@Au@PEG_800_ nanoconjugates left the lungs and increasingly accumulated in the liver. A significantly higher amount of PEG conjugated nanoconjugate was found in the liver in the mice that were not treated with clodronate [Fig. 2(b)]. The same pattern was also seen for the 3500 and 5000 Da PEG nanoconjugates tested. As the PEG linker was lengthened, three phenomena were observed: maximum lung uptake decreased; the difference between clodronate and untreated mice became less pronounced; and spleen uptake increased.

### Immunohistochemistry with anti-thrombomodulin-labeled nanoparticles

Immunohistochemistry showed identical staining patterns for the primary antibody as with the anti-thrombomodulin labeled nanoparticles, demonstrating the persistent antigen recognition and binding of the conjugated antibody [Fig. 2(c)].

### *In vivo* biodistribution of targeted [^177^Lu]Lu_0.5_Gd_0.5_(PO_4_)@Au@PEG_800_@Ab

**FIG. 3.**
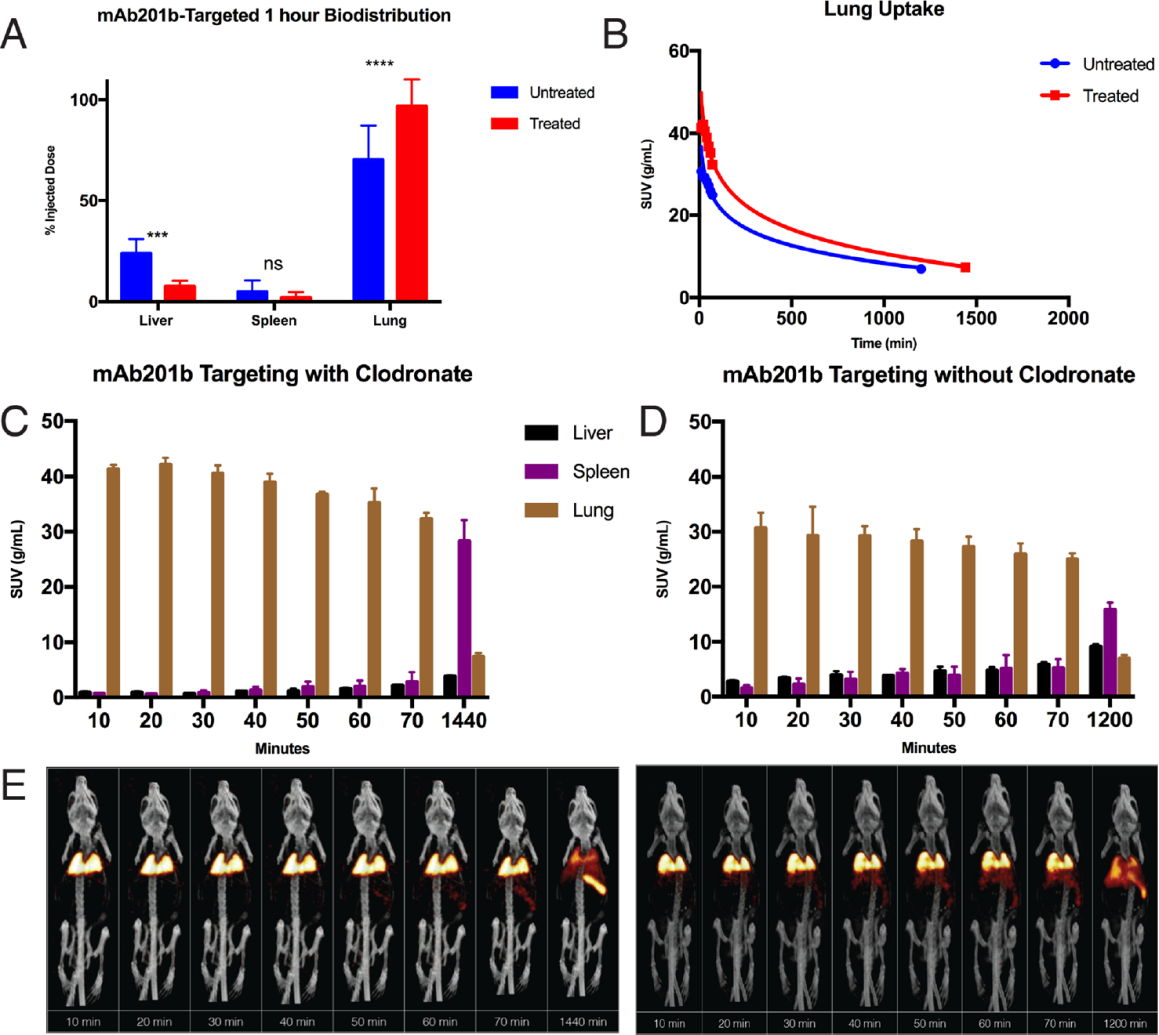
(a) Biodistribution of [^177^Lu]Lu_0.5_Gd_0.5_(PO_4_)@Au@PEG_800_@mAb201b at 1 h post-injection in both clodronate and untreated mice. (***p < .001, by 2-way ANOVA) (b) SUV in lungs over time for mAb201b targeted particles. (c) SUV of [^177^Lu]Lu_0.5_Gd_0.5_(PO_4_)@Au @PEG_800_@mAb201b in mice treated with clodronate and (d) untreated mice. (e) SPECT/CT images of [^177^Lu]Lu_0.5_Gd_0.5_(PO_4_)@Au @PEG_800_@mAb201b over time in mice treated with clodronate (left) and untreated mice (right).

Uptake of mAb-201b labeled nanoconjugates in the lungs is rapid, and the binding is specific. At 10 min post-injection of the nanoconjugates in clodronate-treated mice, more than 95% ID was found in the lungs. This uptake was retained in the lungs for at least 1 h. After 24 h in the clodronate-treated mice, the proportion of the nanoconjugates in the lungs decreased, while the amount in the liver increased slightly and the spleen uptake rose significantly [presumably due to reticuloendothelial system (RES) sequestration]. The redistribution of the nanoconjugates to liver and spleen at 24 h is consistent with previous studies that used mAb-210b labeled nanoparticles.^13,14^ In mice that were not treated with clodronate, there was less uptake in the lungs and increased uptake in the liver and spleen at all time points [Figs. 3(a) and 3(b)].

Depression of the RES by clodronate liposomes increased lung uptake of the nanoconjugates by ∼25% ID and kept liver uptake at less than 10% ID with even less uptake seen in the spleen [Figs. 3(c) and 3(d)]. Single-photon emission computerized tomography (SPECT/CT) images were taken in 10-min intervals for 70 min and imaged again at 20 or 24 h [Fig. 3(e)]. The images clearly show rapid lung uptake of the nanoconjugates in both the clodronate-treated and untreated control mice. However, in the clodronate-treated mice, the uptake was higher and more localized to the lungs than in the controls. In the untreated mice, nanoconjugates accumulated in the liver and spleen within 20–30 min post-injection.

## CONCLUSION

Radiolabeled, mixed core lutetium/gadolinium phosphate nanoparticles have been developed and shown to be highly stable under a variety of conditions. We have demonstrated that the Lu_x_Gd_1-x_(PO_4_) nanoparticles can be synthesized with x varying from 0.1 to 0.9. The addition of a gold coating on the radiolabeled nanoparticles dramatically increased the retention of the radiotracer in the nanoparticle and allowed targeting agents such as the antibody mAb-201b to be successfully conjugated to the surface. Altering the PEG linker chain length used to attach the antibody to the nanoparticle significantly altered the biodistribution of the nanoconjugates *in vivo*. Of the three nanoconjugates used in biodistribution studies, the mAB-201b-labelled nanoconjugate with the shortest PEG linker was selected for *in vivo* lung targeting because it yielded the greatest lung uptake.

Immunohistochemistry demonstrated the persistent specific antigen binding of the mAb-201b antibody when conjugated to the nanoparticle. In the SPECT measurements, uptake of the antibody-labeled nanoconjugates in the lungs was more than 95% ID within minutes after injection in the clodronate-treated mice while non-target organ accumulation was depressed. It has been previously demonstrated in blocking studies that the lung uptake of mAb-201b labeled nanoparticles is mediated by the thrombomodulin receptor by pre-injecting the mice with mAb-201b.^7,13,14^ Competition with unconjugated antibody decreased lung uptake of antibody-labeled nanoparticles (NPs) by a factor of nearly 20.

The RES uptake of the antibody labeled nanoparticles in clodronate-treated mice was substantially delayed, demonstrating the utility of clodronate liposomes in targeting specific antigens. While still not approved in the United States, clodronate is approved for use in humans in Australia, Canada, Italy, the United Kingdom, and several other countries as a bone resorption inhibitor, and it is commonly prescribed for treatment of myeloma and secondary breast cancer.

This work demonstrates that labeling of gold-coated lanthanide phosphate nanoparticles with antibodies can lead to promising anti-cancer theranostic nanoconjugates. The 208 keV gamma ray from ^177^Lu decay (11%) can be used for SPECT imaging of the radiotherapeutic agent, while the moderate energy beta emitted in the decay, as recently demonstrated by Lutathera, can be highly effective in treating metastatic disease. By using 20 mCi of activity in the synthesis, three radioactive atoms would, on average, be incorporated in each nanoparticle (supplementary material). Future work with the nanoconjugates described in this study will involve targeting tumor colonies in a murine metastatic lung disease model with both short-range, high linear-energy transfer alpha particles (^225^Ac) and long-range, low linear-energy transfer beta particles (^177^Lu).

## METHODS

All chemicals were purchased from Sigma-Aldrich and were of ACS grade or better. Ultrapure water with a resistivity of 18.2 MΩ/cm from a Milli-Q water purification system was used to prepare all solutions. A 10 kDa molecular weight cutoff (MWCO) regenerated cellulose dialysis membrane (Spectra/Por 6, 132570) was used for radiochemical yield and stability studies. A 0.4 T NdFeB magnet (Supermagnet #31, United Nuclear) was used to magnetically separate active nanoparticles. Nanoparticles and nanoconjugates were characterized by transmission electron microscopy (TEM, JEOL 1400), neutron activation analysis (NAA), ultraviolet-visible (UV-vis) spectroscopy, and dynamic light scattering (DLS, Malvern zeta sizer, nano ZS).

### [^177^Lu]Lu_0.5_Gd_0.5_(PO_4_) nanoparticle core preparation

Nanoparticle cores with a 50:50 ratio of lutetium:gadolinium were synthesized by mixing 100 *μ*l of 0.05 M LuCl_3_ and 100 *μ*l of 0.05 M Gd(NO_3_)_3_ with 400 *μ*l of 0.05 M sodium tripolyphosphate (Na-TPP) in a 5 ml glass v-bottom vial with a stir bar. The gadolinium in the core allowed the nanoparticles to be magnetically separated, which is useful during subsequent synthesis steps. For the preparation of radioactive nanoparticles, 1–10 mCi of ^177^Lu in 0.1 M HCl (10–20 *μ*l) was added to the solution before heating. The solution was heated for 3 h at 90 °C while stirring slowly. The white solution was then centrifuged at 6000*g* for 5 min to collect the nanoparticle cores. After decanting, the core nanoparticles were redispersed in 1.5 ml of 18 MΩ water. Along with the 50:50 core composition, nanoparticle cores were also synthesized with 90:10 and 75:25 ratios by altering the volume of the lutetium and gadolinium solutions in the reaction mixture.

The core nanoparticles were characterized by transmission electron microscopy (TEM) and neutron activation analysis (NAA). TEM samples were prepared by pipetting 8 *μ*l of a freshly sonicated sample of nanoparticles on a copper grid. The samples were allowed to dry for 5 min, and excess liquid was wicked off with filter paper. The grids were then carbon coated. The coating was necessary because the particles were magnetic. The images were analyzed using ImageJ software available from the National Institutes of Health (NIH). NAA samples were prepared by adding 10 *μ*l of the redispersed nanoparticle solution to a 2/5 dram high-density polyethylene vial and allowed to dry. The vials were sealed and irradiated for 7 s in the University of Missouri Research Reactor pneumatic tube irradiation facility, allowed to decay for 1 min, and then counted for 1 min on a high-resolution gamma spectrometer system.

### [^177^Lu]Lu_0.5_Gd_0.5_(PO_4_)@Au

In order to gold coat the core nanoparticles, the 1.5 ml redispersed solution of nanoparticle cores was transferred to a 5 ml glass v-bottom vial with a stir bar and 600 *μ*l of 0.1 M sodium citrate, and the solution was heated to 90 °C. Next, five 500 *μ*l aliquots of a 2 mM NaAuCl_4_ solution were added dropwise every 5 min (a total of 2.5 ml). During the addition, the solution changed to a deep red color. After heating at 90 °C for an additional 45 min, the solution was transferred to a 20 ml glass scintillation vial and placed next to the 0.4 T NdFeB magnet overnight. The supernatant was decanted, leaving only the magnetically active gold-coated nanoparticles. The approximately 2.6 mg of nanoparticles were then re-suspended in a 2 mM sodium citrate solution. The gold-coated nanoparticles were characterized by TEM, NAA, and UV-vis spectroscopy.

### [^177^Lu]Lu_0.5_Gd_0.5_(PO_4_)@Au@PEG

A polyethylene glycol (PEG) linker was added to the gold-coated nanoparticles for *in vivo* stability and to facilitate the attachment of the antibody. To attach the carboxy-lipoamide PEG (MW = 800, 3400, and 5000 Da, Thermo Scientific), ∼1 mg of PEG was added to 1 mg of gold-coated nanoparticles in 1 ml of 18 MΩ water and stirred at ambient temperature for 4 h. The resulting nanoconjugates were centrifuged at 6000*g* for 10 min and decanted to remove excess PEG. The pegylated nanoparticles were redispersed in 18 MΩ water or phosphate-buffered saline (PBS). The nanoconjugates were characterized using TEM, UV-vis spectroscopy, and DLS.

### [^177^Lu]Lu_0.5_Gd_0.5_(PO_4_)@Au@PEG@Ab

Antibody conjugation was achieved by EDC/NHS activation [1-ethyl-3–(3-dimethylaminopropyl)carbodiimide/N-hydroxysuccinimide]. First, 8 *μ*l of 10 mg/ml of sulfo-NHS and 80 *μ*l of 10 mg/ml of EDC were added to a vial containing 0.5 mg of the radiolabeled Lu_0.5_Gd_0.5_(PO_4_)@Au@PEG_800_ nanoconjugates in 1 ml of PBS buffer (pH = 7.4). After stirring for 30 min, the nanoconjugates were centrifuged at 8000*g* for 5 min, and the supernatant was removed. The nanoconjugates were redispersed in fresh PBS buffer and again centrifuged and decanted to remove unreacted sulfo-NHS and EDC to prevent antibody crosslinking. Once redispersed in PBS, 0.5 mg of antibody was added to the vial and the solution was placed on a rocker table and allowed to mix overnight. The nanoconjugates were then centrifuged, and the supernatant, containing unreacted antibody, was removed. The nanoconjugates were then redispersed in PBS. Previous work has demonstrated that the antibody to nanoparticle ratio saturates at four at these molar ratios.^7^

### Immunohistochemistry of anti-thrombomodulin-labeled nanoparticles

The EDC/NHS activation method to conjugate antibodies is by nature nonspecific. To determine if the antibody retained its specific antigen-binding ability once conjugated to the nanoparticle surface, immunohistochemistry was performed on formalin-fixed normal human lung tissue samples. Nanoparticles were labeled with anti-thrombomodulin antibody as described above. Staining was performed using a Dako K4065 Envision Dual Link Kit utilizing horseradish peroxidase (HRP) with diaminobenzidine (DAB) as the final chromogen. Formalin-fixed paraffin-embedded 6 *μ*m sections of normal human lung tissue were deparaffinized with xylene, rehydrated through a graded series of ethanol (100%, 95%, and 90%), and then were rinsed with distilled water.

As positive controls, the primary antibody was incubated on the sections following antigen retrieval overnight in a humidity chamber at 4 °C, after blocking endogenous peroxidase with the Dako dual endogenous block solution. The antibody (Abcam ab6980, Cambridge, MA) was used at a 1:250 dilution. Following the overnight incubation, the slides were brought to room temperature, rinsed gently with buffer for 5 min, then covered with the kit's labeled polymer linked to HRP, and incubated in the humidity chamber at room temperature for 30 min. This was followed by a 2 min incubation with DAB, and the reaction was stopped by rinsing with running distilled water. Following rinsing, the slides were counterstained with Modified Mayer's Hematoxylin (Newcomer Supply, catalogue #1202) for 1 min, rinsed with tap water, dehydrated through a graded series of ethanol (95% ×2, 100% ×2) followed by xylene, and coverslipped with Leica Surgipath MM24 mounting medium.

To test the antibody conjugated nanoparticles, the suspension of nanoparticles prepared as described was used as a substitute for the primary antibody; all other procedures were identical. Negative controls utilized no primary antibody.

### Radiochemical yield and stability

The percentage of ^177^Lu incorporated into the nanoparticle cores was determined using a 10 kDa dialysis membrane. After the core synthesis was completed, the nanoparticle solution was loaded into the dialysis membrane and placed in 500 ml of 18 MΩ H_2_O overnight. The next day a 10 ml aliquot of the dialysate solution was analyzed by gamma spectroscopy. After decay correction, the amount of ^177^Lu that leeched out of the dialysis membrane was measured. For the radiochemical yield, the supernatant was decanted from the magnetically collected sample, and the activity in the nanoparticles was determined by gamma spectroscopy. The yield was determined by decay correction and comparison to the original activity of the solution prior to magnetic separation of the nanoparticles.

Stability studies of the gold-coated nanoparticles were also performed using a 10 kDa dialysis membrane. The radioactive gold-coated nanoparticles were loaded into the dialysis membrane and challenged with different solutions. The stability was tested against 500 ml of 18 MΩ H_2_O, PBS buffer (pH = 7.4), and a 50% fetal bovine serum (FBS) solution. A 10 ml aliquot of the dialysate was taken periodically and analyzed by gamma spectroscopy to measure the amount of ^177^Lu that leeched out.

### *In vivo* biodistribution of PEG coated nanoparticles

All biodistribution animal experiments were performed according to the standards of the University of Missouri Institutional Animal Care and Use committee under Protocol #8390. Clodronate liposomes were acquired from ClodronateLiposomes.org. Organ specimens were weighed and then counted using a calibrated sodium iodide (NaI) well detector to detect the 208 keV (11%) gamma emission from ^177^Lu.

Three groups of ten C57BL/6 mice were injected intravenously via the tail vein with [^177^Lu]Lu_0.5_Gd_0.5_(PO_4_)@Au@PEG_800_, [^177^Lu]Lu_0.5_Gd_0.5_ (PO_4_)@Au@PEG_3400_, or [^177^Lu]Lu_0.5_Gd_0.5_ (PO_4_)@Au@PEG_5000_, in a suspension of 5 mg/ml of bovine serum albumin (BSA) in PBS. In each case, a small amount of material was observed to settle out in the NP/BSA/PBS solution over time. Prior to injection, the nanoparticles were re-dispersed in solution with simple vortex mixing. In each of the three groups, half of the mice were injected with clodronate liposomes (40 mg/200 *μ*l) intraperitoneally 72 h before nanoconjugate injection in order to suppress the reticuloendothelial system (RES) and increase the blood circulation time of the nanoconjugates.^15,16^ The biodistribution was determined at 1 h and 24 h post-injection. A fourth group of 5 control mice was injected with [^177^Lu]Lu_0.5_Gd_0.5_ (PO_4_)@Au without any surface modification. Mice were euthanized by intraperitoneal injection of pentobarbital (200 mg/kg).

### *In vivo* lung targeting with mAb-201b labeled [^177^Lu]Lu_0.5_Gd_0.5_(PO_4_)@Au@PEG_800_@Ab

All the following animal studies were performed in accordance with the Animal Care Committee (ACC) of the University of British Columbia under the approved protocol A16-0150. Two groups of three BALB/c mice (Charles River Laboratories) were anesthetized using isoflurane inhalation and received a subcutaneous injection of lactated Ringer's solution (0.5 ml) for hydration prior to the imaging experiment. Approximately 300 *μ*Ci of mAb-201b labeled [^177^Lu]Lu_0.5_Gd_0.5_(PO_4_)@Au@PEG_800_@Ab nanoconjugates were administered via tail vein injection. Group 1 was used as control (untreated mice), whereas group 2 received intraperitoneal administration of 100 *μ*l clodronate liposomes 72 h prior to nanoconjugate injection. Whole-body SPECT/CT scans were acquired at 10, 20, 30, 40, 50, 60, 70 min, and 20 h postinjection for group 1. Group 2 was imaged at 10, 20, 30, 40, 50, 60, 70 min, and 24 h post-injection. Both groups were euthanized by perfusion fixation under terminal anesthesia immediately following the last imaging time point. After collecting the organs, the radioactivity in the murine lungs and livers was measured using a well counter (Biodex Atomlab 500). All imaging was performed using a Vector SPECT/PET-CT scanner, MILabs (Utrecht, the Netherlands), equipped with a high-energy collimator. Following each SPECT acquisition, a whole body CT scan was performed to obtain anatomical information and the images were co-registered. Throughout the entire scanning procedure, mice were kept under isoflurane anesthesia and constant body temperature was maintained using a heating pad. Images were reconstructed using the standard manufacturer supplied software after correction for scatter and attenuation. Data were expressed in terms of radioactivity concentration using a well counter-derived calibration factor. Hand-drawn regions of interest (ROI) were placed on the images of the lungs, spleen, and liver. Standard uptake values (SUV), corresponding to the concentration in the region of interest/injected activity/body weight, were estimated for each organ at each time point. In addition, a %ID for each organ was estimated by evaluating the total radioactivity in each organ and dividing it by the injected dose.

## SUPPLEMENTARY MATERIAL

See supplementary material for the results of the radiochemical stability studies and the calculation of the average number of radioactive atoms per nanoparticle.
